# Impact of fullerol C_60_(OH)_24_ nanoparticles on the production of emerging toxins by *Aspergillus flavus*

**DOI:** 10.1038/s41598-020-57706-3

**Published:** 2020-01-20

**Authors:** Tihomir Kovač, Ivana Borišev, Marija Kovač, Ante Lončarić, Frane Čačić Kenjerić, Aleksandar Djordjevic, Ivica Strelec, Chibundu N. Ezekiel, Michael Sulyok, Rudolf Krska, Bojan Šarkanj

**Affiliations:** 1 Josip Juraj Strossmayer University of Osijek, Faculty of Food Technology, Department of Applied Chemistry and Ecology, Franje Kuhača 20, 31000 Osijek, Croatia; 2 Institute of Bioanalytics and Agro-Metabolomics, Department of Agrobiotechnology (IFA-Tulln), University of Natural Resources and Life Sciences Vienna (BOKU), Konrad Lorenzstr. 20, 3430 Tulln, Austria; 3 University of Novi Sad, Faculty of Sciences, Department of Chemistry, Biochemistry and Environmental protection, Trg Dositeja Obradovića 3, 21000 Novi Sad, Serbia; 4 Inspecto Ltd., Industrijska zona Nemetin, Vukovarska cesta 239b, 31000 Osijek, Croatia; 5 Department of Microbiology, Babcock University, Ilishan Remo, Ogun State Nigeria; 6 Institute for Global Food Security, School of Biological Sciences, Queen’s University Belfast, University Road, Belfast, BT7 1NN Northern Ireland, United Kingdom; 7 University North, Trg dr. Žarka Dolinara 1, 48000 Koprivnica, Croatia

**Keywords:** Nanoparticles, Nanoparticles, Environmental impact, Environmental impact

## Abstract

The impact of fullerene C_60_ water soluble daughter molecules - fullerols C_60_(OH)_24_ nanoparticles (FNP) on emerging (non-aflatoxin biosynthetic pathway) toxins production in mycelia and yeast extract sucrose (YES) media of *A. flavus* was investigated under growth conditions of 29 °C in the dark for a 168 h period. The FNP solution (10, 100 and 1000 ng mL^−1^) contained predominantly nanoparticles of 8 nm diameter and with zeta potential mean value of −33 mV. Ten emerging metabolites were produced at concentrations reaching 1,745,035 ng 50 mL^−1^ YES medium. Seven of the metabolites were found in mycelia and media, while three were only in mycelia. Majority of the metabolites were detected in higher quantity in mycelia than in media, at a ratio of 99:1 (*m/m*). However, higher metabolite quantities were found in media following FNP application, while FNP caused a decrease of total metabolite quantities in mycelia. The concentrations of the metabolites in media increased in the presence of 1000 ng mL^−1^ FNP while mycelial quantities of the metabolites decreased with increased applied FNP dose. The impacts of global climate changes on FNP availability in the environment and on mycotoxin occurrence in crops increase the relevance of this study for risk assessment of nanoparticles. Cordycepin is reported for the first time as metabolite of *A. flavus*.

## Introduction

Global climate changes contribute to increased mycotoxin contamination of foods by shaping the mycotoxigenic fungal community structure in the environment. The most obvious example is the prediction of Battiliani *et al*.^1^ about aflatoxins becoming a food safety issue in Eastern Europe, the Balkan Peninsula and Mediterranean regions in the next 100 years as a result of +2 °C environmental temperature change. Several review papers on the impacts of interacting climate change factors on growth and mycotoxin production by major foodborne fungi have been published^2–8^. The most important mycotoxigenic fungi is *Aspergillus flavus*, producer of the strongest known natural hepatocarcinogen – aflatoxin B_1_ (AFT B_1_). This fungus has been widely studied due to the severe health and economic challenges it causes through aflatoxin contamination of foods. The dynamics of *A. flavus*, influenced by factors of global climate changes (e.g. temperature, drought stress and CO_2_ concentration) have also been recently studied^2,9,10^. Besides the regulated mycotoxins, the impact of climate change on the emerging toxins are receiving increasing attention in recent years^2,11^. According to Kovalsky *et al*.^11^ emerging toxins are a group of chemically diverse mycotoxins for which to date no regulations exist. For example, the AFT precursors are one of the emerging toxins group^11,12^ and there is only limited literature available on the emerging toxins from *Aspergillus* species^11,13^. For the purpose of the present study, emerging toxins produced by *A. flavus* are the non-aflatoxin biosynthetic pathway metabolites.

Similar to the attention global climate changes received in the past two decades^1,14^, nanotechnology research, development and application have also skyrocketed. Increasing the application of nanoparticles to various commodities raises chances for environmental release during production processes, use or disposal of products. Consequently, the utilization of nanoparticles should be based on thorough knowledge of their effects on biological systems as well as on the aforementioned abiotic stressors^15,16^. The most intensely investigated carbon-based nanoparticles are fullerenes and their hydroxylated derivatives due to their numerous applications^17–19^. The major side effect of their usage is the determined realistic environmental occurrence of fullerene C_60_ in almost every part of the environment (wastewaters, surface waters, river sediments and soils)^20–22^. In addition, the increase of fullerenes in sewage sludge-treated soil on annual basis was predicted by Sun *et al*.^23^. Fullerene C_60_ can, however, be spontaneously mineralized into water soluble daughter molecules (fullerols C_60_(OH)_24_ (FNP)) over 16 weeks in the environment^24^.

Despite the over 20-year long research period, the environmental reactivity of fullerene C_60_ and FNP is still poorly defined, mainly due to distinctive material properties and biological activities such as induction of side effects on cells, organelles and biomolecules^19,25–32^. The interaction between FNP and mycotoxigenic fungi (e.g. *A. flavus*) has been under research but literature are still limited^13,33,34^. However, it is known that FNP modulates oxidative status of *A. flavus*, consequently affecting the production of aflatoxins and their precursors^13,34^. Medina *et al*.^9,10,35^ reported stimulation of AFT B_1_ production by fluctuations in environmental abiotic stressors and secondary metabolites regulatory gene shifts. According to that report, the mechanism of FNP biological activity related to antioxidative properties could play a role in oxidative status perturbations in the sensitive saprophytic soil fungus, *A. flavus*, during FNP mineralization process.

Therefore, the aim of this study was to determine secondary metabolite production besides aflatoxins and their precursors in the mycelia and growth media of *A. flavus*, and to confirm whether there are secondary metabolite shifts in the *A. flavus* cells under FNP influence. This is one of the follow up studies focused on interaction of FNP with mycotoxigenic fungi and is one step closer to determination of mechanism of FNP action.

## Results and Discussion

The geographical distribution of mycotoxigenic fungi determines mycotoxin contamination worldwide, and this is a reflection of environmental temperature, humidity and CO_2_ concentration^35^. With the predicted changes in climate expected to occur in many parts of the world including eastern Europe, it is of necessity to understand the role of neglected environmental compounds (e.g. the abiotic stressor – FNP) in the production of emerging toxins by *A. flavus*. The FNP solution (10 µg mL^−1^) used in this study was obtained from the same batch of FNP solution prepared, characterised and used in the study performed by Kovač *et al*.^13^ and the final concentrations present in YES media were 10, 100 and 1000 ng mL^−1^. The concentrations of 10 and 100 ng mL^−1^ of FNP are present in environment at this moment^20–22^, while occurrence of 1000 ng mL^−1^ is expected in the future. Consequently and for the reasons stated by Kovač *et al*.^34^, we wanted to examine what the effect of possible future environmental concentration spike reaching about 1000 ng/mL could result in. However, we also included the lower (10 ng/mL) and present high (100 ng/mL) concentrations in this study.

As previously reported, the FNP solution contained only particles smaller than 100 nm (range: 5–25 nm). The particles had a hydrodynamic radius of 8 nm and the zeta potential of the FNP aqueous solution was –33 mV. The particle diameter is in accordance with EC Recommendation for the definition of the term “nanomaterial”^36^.

**Figure 1:**
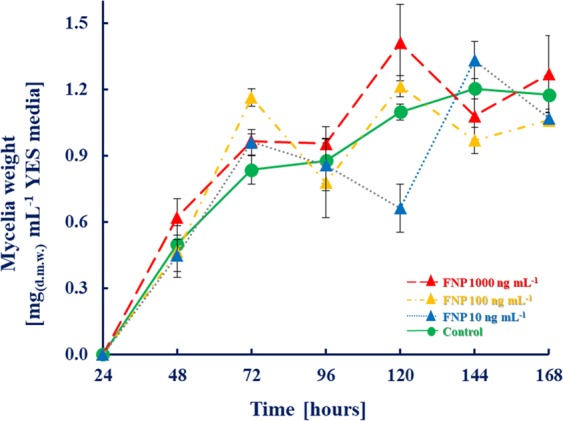
Fullerenol C_60_(OH)_24_ nanoparticles (FNP) influence on *A. flavus* NRRL 3251 biomass production (expressed as mg of dry weight (mg.d.w.) per mL of media) in YES medium incubated over a 168 h period at 29 °C. Data represent the mean ± SEM from three separate experiments.

Figure 1. depicts the effect of FNP on *A. flavus* biomass production. Overall, the results agree with previously reported data on FNP influence on *A. flavus* biomass production^13^. At least 48 h incubation period was required to obtain measurable quantity of mycelia in the first instance and in this study, there was no statistically significant (p > 0.05) effect of the applied concentrations of FNP on biomass production at all the time intervals^13^. The fact that growth of fungi under abiotic stressor remains practically unaffected is in accordance with results of Medina *et al*.^9,10^ and Kovač *et al*.^13^.

Regarding the effect of FNP on metabolite/toxin production by *A. flavus*, we did not investigate in this study the effect on production of AFT B_1_ and its precursors, which are regarded as the main metabolites from *A. flavus*. Detailed explanation on FNP effect on AFT B_1_ and precursor metabolites are given in our previous report Kovač *et al*.^13^. From that report, FNP exerted a concentration-dependent effect on the AFT biosynthesis pathway metabolites and altered sterigmatocystin (ST) export from the cell. Prior to 120 h of growth of *A. flavus*, FNP exerted antioxidative potential, which vanished afterwards to cause strong and concentration-dependent rise in biosynthesis of aflatoxins. However, it is pertinent that we investigated, in this present study, the possibility of FNP modulation of emerging toxins production in *A. flavus* mycelia and growth media. Mostly due to conclusions of Medina *et al*.^35^ who suggested that abiotic stressors could stimulate AFT B_1_ production owing to regulatory shifts in AFT B_1_ and cyclopiazonic acid gene clusters, and our understanding that FNPs are behaving as the classical abiotic stressors, but belong to the group of the new abiotic stressors.

**Figure 2:**
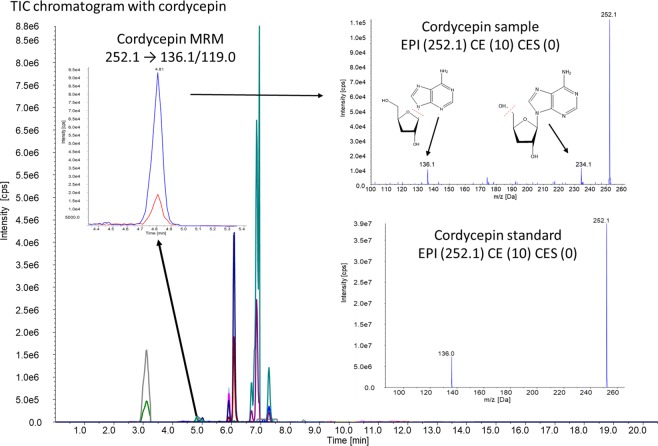
Total ion chromatograms of the samples containing cordycepin with the MRM transition, and EPI scan compared to the EPI scan of the pure standard. Cordycepin MRM diagram – *y* axis: Intensity [cpc] from 5000 to 9.5e4, *x* axis: Time [min] from 4.4. to 5.4; Cordycepin sample diagram – *y* axis: Intensity [cpc] from 1.0e4 to 1.1.e5, *x* axis: m/z [Da] from 100. to 260; Cordycepin standard diagram – *y* axis: Intensity [cpc] from 0 to 3.9e7, *x* axis: m/z [Da] from 100. to 260.

**Figure 3:**
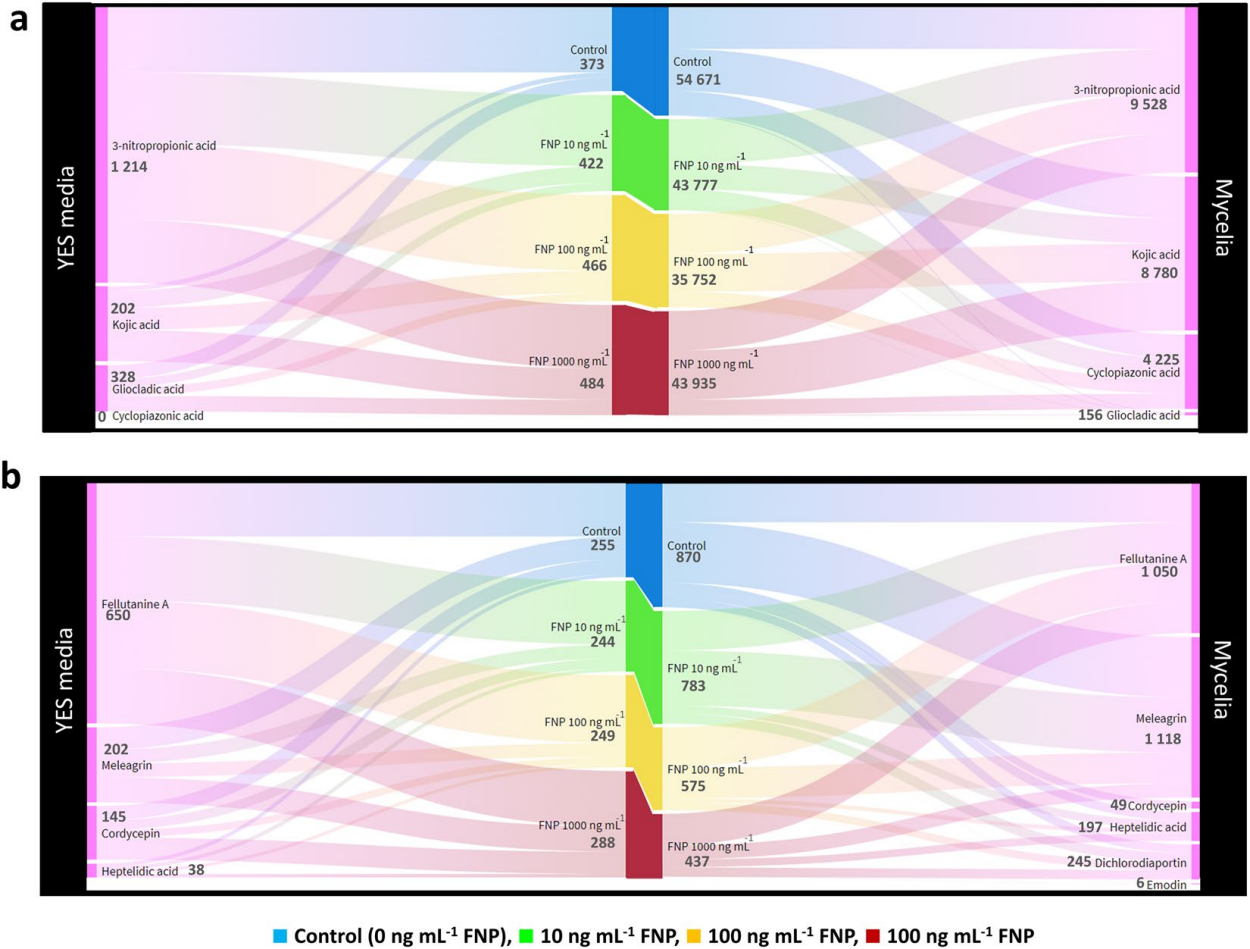
*Aspergillus flavus* NRRL 3251 emerging toxins (a – major mycotoxins; b – minor mycotoxins) distribution in mycelia and growth media during growth in YES medium after 168 h at 29 °C influenced by fullerenol C_60_(OH)_24_ nanoparticles (FNP). Data represent the mean from three separate experiments and are expressed in µg 50 mL^−1^ for major metabolites (**a**) and ng 50 mL^−1^ for minor metabolites (**b**). The flow thickness represents the relative concentration ratio between metabolites. The numbers near applied FNP concentrations are representing the sum of produced mycotoxins, both at (**a**,**b**). The numbers by names of mycotoxins are representing the sum of produced mycotoxins (ng mL^−1^) at all tested FNP concentrations, both for (**a**,**b**).

In this study, the emerging *A. flavus* toxins fellatunine A, gliocladic acid, heptelidic acid, meleagrin, kojic acid, cordycepin, 3-nitropropionic acid, cyclopiazonic acid, emodin and dichlorodiaportin were detected. Cordycepin is reported here for the first time as metabolite of *Aspergillus*, specifically *A. flavus*; chromatograms and EPI fragmentation scans are given in Fig. 2. All aforementioned emerging toxins were detected in both mycelia and growth media, with the exception of cyclopiazonic acid, emodin and dichlorodiaportin, which were found only in mycelia (Fig. 3). The 10 metabolites detected in this study were categorized as minor (fellatunine A, heptelidic acid, meleagrin, cordycepin, emodin and dichlorodiaportin) and major (gliocladic acid, kojic acid, 3-nitropropionic acid and cyclopiazonic acid) based on their production at <1000 ng 50 mL^−1^ and >1000 ng 50 mL^−1^ levels, respectively.

**Figure 4:**
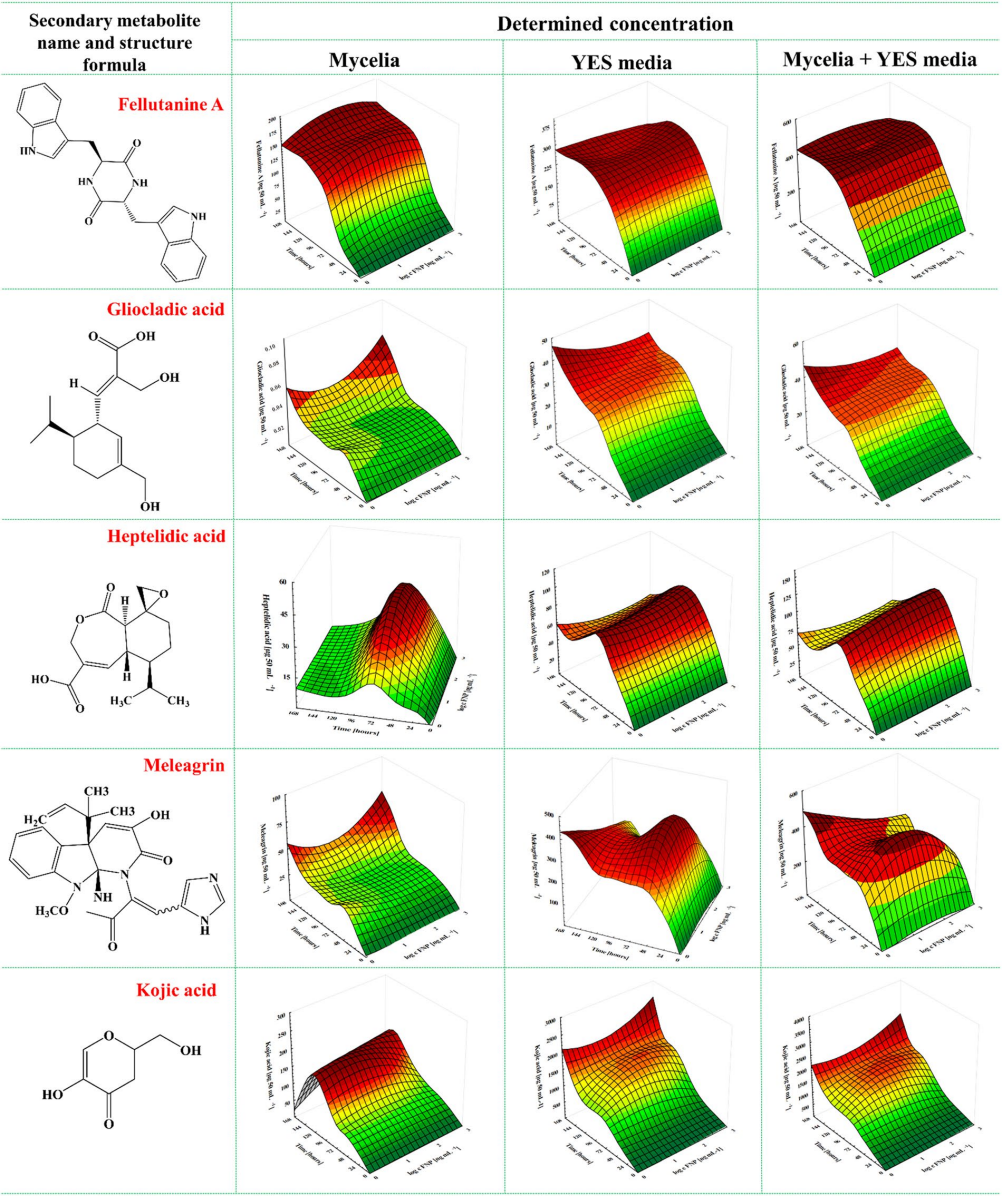
Fullerenol C_60_(OH)_24_ nanoparticles (FNP) influence of on *Aspergillus flavus* NRRL 3251 emerging toxins biosynthesis - fellatunine A, gliocladic acid, heptelidic acid, meleagrin and kojic acid during *A. flavus* growth in YES medium for 168 h at 29 °C. Data represent the mean ± SEM from three separate experiments.

Fellutanine A is a naturally occurring 2,5-diketopiperazines, bio-active diketopiperazine alkaloid obtained from species of several fungal genera^37,38^. This metabolite was found in higher quantity in growth media than in *A. flavus* mycelia (Fig. 3). There were no statistically significant changes in fellutanine A concentration under FNP presence, neither in growth media nor in mycelia (Fig. 4). In addition, gliocladic acid and heptelidic acid were detected both in mycelia and growth media, with most of the metabolites remaining in YES media (67.7% gliocladic acid) and mycelia (83.8% heptelidic acid) (Fig. 3). These metabolites are known to be produced by species of several fungal genera including *Aspergillus*, *Chaetomium*, *Gliocladium* and *Trichoderma*^39^. Similar to fellutanine A, FNP did not significantly affect gliocladic acid biosynthesis at any of the tested concentrations. However, there was statistically significant difference (p = 0.02) in heptelidic acid concentrations between control samples and samples treated with 100 ng mL^−1^ FNP (Fig. 4).

The fungal secondary metabolite meleagrin, which possesses neurohumoural and antibiotic activity^40^, was quantified in mycelia and growth media, with higher concentrations in the mycelia than in the growth media (Fig. 4). Statistically significant (p = 0.04) increase in the meleagrin concentration (up to 10%) was recorded in growth media containing 1000 ng mL^−1^ FNP at 168 h of fungal growth in comparison to the control samples.

**Figure 5:**
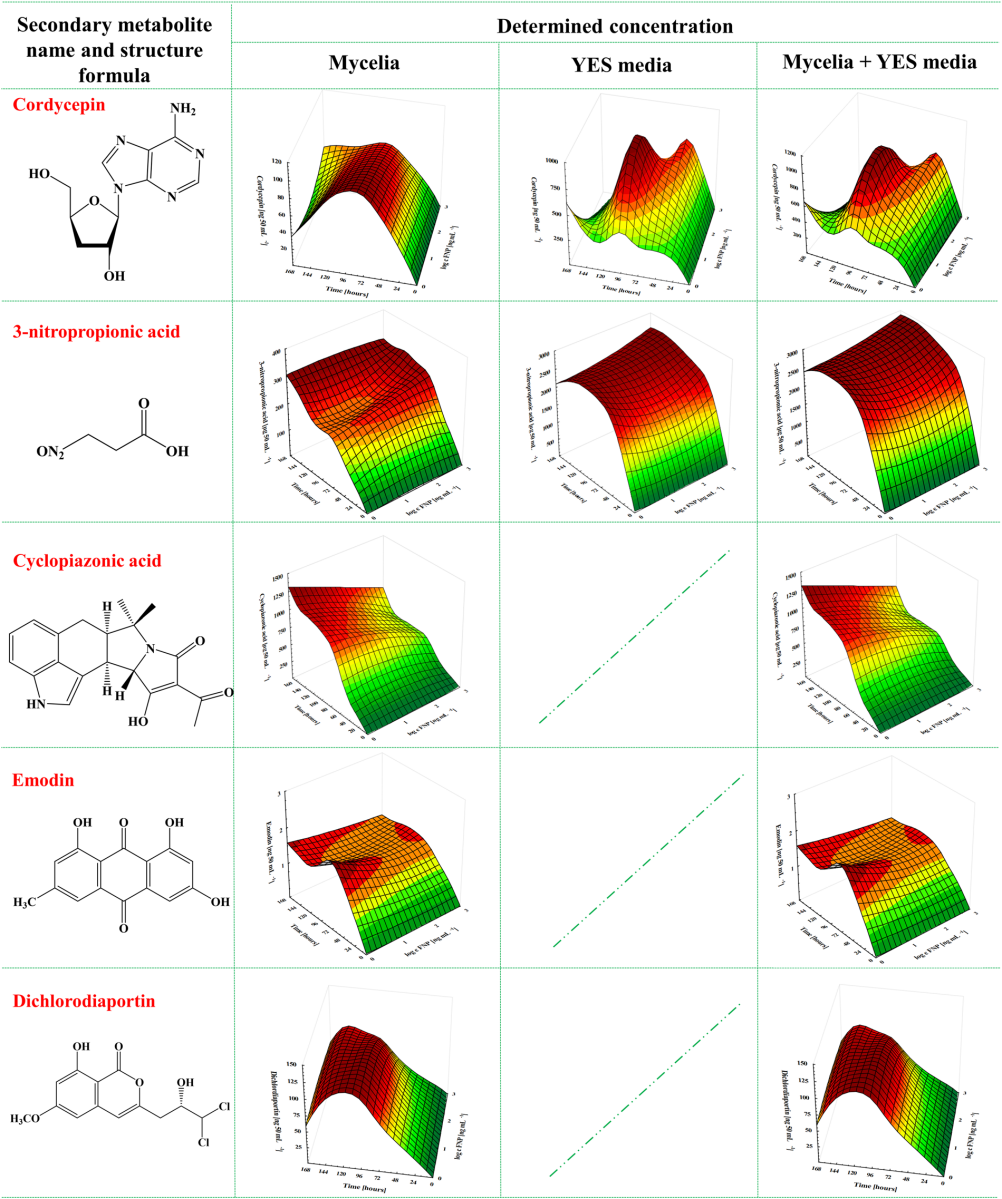
Fullerenol C_60_(OH)_24_ nanoparticles (FNP) influence of on *Aspergillus flavus* NRRL 3251 emerging toxins biosynthesis – cordycepin, 3-nitropropionic acid, cyclopiazonic acid, emodin and dichlorodiaportin during *A. flavus* growth in YES medium for 168 h at 29 °C. Data represent the mean ± SEM from three separate experiments.

Cordycepin is considered an immunological regulator, anticancer, antifungal, antiviral, antileukemia and antihyperlipidemia agent^41^. This metabolite, also regarded as a Phase I/II clinical stage drug candidate for the treatment of refractory acute lymphoblastic leukemia patients who express the enzyme terminal deoxynucleotidyl transferase^41^ was one of the emerging metabolites produced in higher amount in YES media (74.7%) than in mycelia, although the difference between mycelia and media concentrations were statistically insignificant (Fig. 5). Specifically, the cordycepin concentration quantified in YES media supplemented with 1000 ng mL^−1^ FNP and incubated for 168 h was the highest (34.5%) compared to other FNP concentrations (Fig. 3b). Kojic acid was also detected in both mycelia and media, albeit at higher concentrations in mycelia (96.4%) than in the YES media. The concentration of kojic acid in YES media was, however, elevated with an increase in the FNP concentration (72.6% at 10 ng mL^−1^ FNP, 78.6% at 100 ng mL^−1^ FNP and 84.2% at 1000 ng mL^−1^ FNP) at 168 h of growth (Figs. 3a and 4). Kojic acid is known to be produced by *Aspergillus* spp., *Penicillium* spp. as well as some other fungi, and is a tyrosinase activity inhibitor, food additive, skin-whitening agent, antioxidant, anti-tumour and radioprotective agent^42^. 3-nitropropionic acid, a major secondary metabolite specific to Aspergilli, was detected both in mycelia and media, although the mycelial concentration was higher (88.7%) than the concentration in the growth media (Figs. 3a and 5). Statistically significant difference (p = 0.03) was recorded for the sum of 3-nitropropionic acid concentrations produced in mycelia and media when *A. flavus* was grown in YES media containing 10 and 1000 ng mL^−1^ FNP at 120 h (Fig. 5).

In addition to the metabolites found in both mycelia and media, three metabolites (cyclopiazonic acid, emodin and dichlorodiaportin) were detected only in the mycelia. Cyclopiazonic acid is known to react with ambient oxygen, causing its degradation^43^. During the incubation of the media under shaking conditions, contact between oxygen and the media is increased, causing the degradation of cyclopiazonic acid which has been released into the media^43^. Consequently, only cyclopiazonic acid retained in the mycelia without direct contact with oxygen remains stable and easily detected; this is the most logical reason why these three metabolites were not found in the media. Many ascomycetous fungi belonging to *Aspergillus* spp. and *Penicillium* spp. produce cyclopiazonic acid, which causes degenerative changes and necrosis in the liver, spleen, pancreas, kidney, salivary glands, myocardium and skeletal muscles, based on toxic effects observed in male and female rats^44^. Aflatoxins often co-occur with cyclopiazonic acid in high quantities in maize and peanuts suggesting that synergism from their co-exposures may occur^44^. In addition, cyclopiazonic acid may be co-produced with other metabolites as found in the present study (Figs. 3–5). The FNP concentrations tested in this study caused a decrease in cyclopiazonic acid concentrations in the amended media compared to the control, with statistically significant differences recorded only at 96 h (p = 0.01) and 144 h (p = 0.01) of growth (Fig. 5). Similar FNP dose-dependent decrease that was reported for AFT B_1_ in YES media^13^ was recorded in our present study. This could be additional data to confirm previous reports of co-occurrence of cyclopiazonic acid with aflatoxins^44^.

Emodin is a fungal secondary metabolite that exhibits diverse biological activities including anticancer and anti-inflammatory functions^45,46^. In *A. flavus* mycelia, emodin was present in low concentrations (0.64–2.53 ng 50 mL^−1^) (Fig. 3b**;** Fig. 4). However, 1000 ng mL^−1^ FNP caused a significant (p = 0.03) decrease in emodin concentration at 72 h of growth. Dichlorodiaportin belongs to the isocoumarins, a group of natural products with diverse chemical structures and pharmacological activities including antibacterial activity^47^. It is reported to be produced a variety of species of fungal genera including *Aspergillus*^47–49^. The highest dichlorodiaportin concentrations were detected at 120 h of growth (Fig. 4), afterwards the concentrations decreased in both control and FNP treated samples.

In general, majority of the *A. flavus* emerging metabolites were determined in higher quantity in mycelia than in YES media at a ratio of 99:1. In YES media higher metabolite quantities were obtained after FNP was applied (11.6% at 10 ng mL^−1^ FNP, 20% at 100 ng mL^−1^ FNP and 22.9% at 1000 ng mL^−1^ FNP), while in mycelia the opposite effect was observed. The presence of FNP during the incubation period caused decrease of total metabolites (24.9% at 10 ng mL^−1^ FNP, 52.9% at 100 ng mL^−1^ FNP and 24.4% at 1000 ng mL^−1^ FNP) (Fig. 3a). When the minor secondary metabolites were compared, a decrease of 4.5 and 2.4% in the concentrations in YES media was recorded under the presence of 10 and 100 ng mL^−1^ FNP, respectively, while an increase of 11.5% was observed at 1000 ng mL^−1^ FNP. When the quantities of minor metabolites in mycelia were compared, a decrease with increased dose of FNP was recorded (10.0% at 10 ng mL^−1^ FNP, 33.3% at 100 ng mL^−1^ FNP and 49.8% at 1000 ng mL^−1^ FNP); this similar trend was recorded also for the major ones (Fig. 3b).

The increased FNP levels can be expected in the future as a consequence of increased production, usage, release, and time-lag in the mineralisation processes in the environment^17–23^. Consequently, according to trends of increasing environmental occurrence of FNP, shaping of the *Aspergillus* spp. community by climate changes and here presented results, it seems that FNPs are capable of posing higher threat levels to the toxigenic potentials of *A. flavus*; this may further be even more aggravated by future climate changes. This study confirmed the ability of FNP to modulate production of 10 emerging *A. flavus* toxins. As previously established, the relevant parameters for environmental risk estimation were data for the sum of the secondary metabolites detected in mycelia and in growth media. FNP in this study exerted a concentration-dependent effect on the production of *A. flavus* emerging toxins. Although some of the metabolites (e.g. cyclopiazonic acid) decreased in concentration with increased dose of FNP, the sum of all concentrations increased. It can be concluded that the antioxidative potential of FNP, which vanishes over time, causes concentration-dependent rise in the sum of secondary metabolites produced by *A. flavus*. Such prospects of FNP occurring in the environment opens up further investigations into its modulation of regulated and emerging toxins in food samples, as well as a study into the photosensitized FNP action on available mycotoxigenic fungal species in other to control mycotoxins occurrence and avoid food safety and human health risks. In this paper, we further report the production of cordycepin in YES media; which was not previously reported in *A. flavus* in available literature.

To sum up above mentioned, the FNP environmental concentration are increasing since production and release are faster than degradation into environment. At the same time, dynamics of *A. flavus* is influenced by temperature, drought stress and CO_2_ concentration due to global climate changes. Accordingly, probability of FNP for interaction with fungi is only increasing and which can certainly be reflected in the amount of secondary metabolites produced, which is confirmed by the results of this study (Figs. 3–5). Moreover, FNP present in environment only can be added value to already known negative effects of mentioned abiotic stressors that cause increase of mycotoxins occurrence. However, this study is one of the follow up studies and for establishing of general opinion of mechanisms of FNP action and their impact on environment, or consequently on food and feed safety, as well as human health, more research is needed.

## Future Perspectives

This report increases our understanding of the different metabolites that *A. flavus* can produce during competition in its environment to exclude other species. Further research may attempt to silence the expression of genes responsible for AFT biosynthesis in favour of genes for codycepin production since cordycepin offers to be of health benefits due to its anticancer, antiviral, antileukemia and antihyperlipidemia activities. Furthermore, this paper has provided data to establish the foundation for further studies on determining the mechanism of FNP action during interaction with mycotoxigenic fungi.

## Materials and Methods

### Chemicals

Yeast extract, potato dextrose agar, malt extract agar and sucrose were purchased from Biolife (Italy). AFT standard mix (B_1_, G_1_, B_2_, G_2_) was purchased from Biopure (Austria). Acetonitrile and methanol (HPLC grade both) were obtained from Merck (Germany). Ammonium acetate and glacial acetic acid (p.a.) were purchased from Sigma Aldrich (Vienna, Austria). For ultrapure water preparation, a Purelab Ultra system (ELGA LabWater, Celle, Germany) was used. Standards of *A. flavus* metabolites were collected from various research groups or purchased from the following commercial sources: Romer Labs^®^Inc.(Tulln, Austria), Sigma–Aldrich (Vienna, Austria), Iris Biotech GmbH (Marktredwitz, Germany), Axxora Europe (Lausanne, Switzerland) And LGC Promochem GmbH (Wesel, Germany). Purchased standards were prepared according to Malachova *et al*.^50^.

### Fullerol C_60_(OH)_24_ synthesis, preparation and characterisation of nanoparticles solution

The synthesis of fullerol C_60_(OH)_24_^49^,preparation of nanoparticle solution in ultrapure water and characterisation of the particles by Dynamic Light Scattering (DLS) and Electrophoretic Light Scattering (ELS) techniques were performed as previously described by Kovač *et al*.^13^. The hydrodynamic size and the surface charge (zeta potential (ζ)) of the nanoparticle solution samples were determined by Zetasizer Nano ZS instrument (Malvern Instruments Inc., UK). All DLS analysis (633 nm wavelength and a measurement angle of 173° (*backscatter detection*)) were performed in triplicates in aqueous solution at ambient temperature (25 °C) while zeta potential (ζ) measurements were performed in duplicates.

### Cultivation of *Aspergillus flavus* on mycological media

*Aspergillus flavus* NRRL 3251 culture maintained on malt extract agar (Biolife, Italy) at 4 °C was used in this study. The *A. flavus* NRRL 3251 strain was grown on potato dextrose agar (Biolife, Italy) in the dark at 29 °C for 7 days to stimulate conidia production. Yeast extract sucrose (YES) broths amended with 0, 10, 100 and 1000 ng mL^−1^ of C_60_(OH)_24_ were prepared. The preparation of fungal conidia suspension, its inoculation into nanoparticle-amended aflatoxin-inducing YES broth in 250 mL flasks and incubation were conducted in the dark at 29 °C, which favours AFT production, as previously described^34^. The rotary shaker (KS 260 basic, IKA, Germany) set at 200 rpm was used for the incubation of the inoculated flasks for 168 h. Every 24 h from the 48^th^ to 168^th^ h of incubation, samples of medium and mycelia were collected from the flasks. Mycelia were separated from the media by filtration and stored in 2 mL vials at −80 °C for at least 24 h until lyophilisation (Christ, Alpha 1-4 LD, Germany). Drying conditions were as follows: freezing temperature −55 °C; temperature of sublimation −35 to 0 °C; vacuum level 0.220 mbar. The temperature of isothermal desorption varied from 0 to 22 °C under the vacuum of 0.060 mbar. Freeze-drying lasted until the constant mass of mycelia was obtained, which was approximately 5 h. Additionally, a portion of the mycelia was taken prior to −80 °C storage and lyophilisation, and dried until constant mass (24 h at 105 °C) in order to determine the dry mycelial weight.

### Determination of emerging toxins in mycelia and culture media of *A. flavus*

The metabolites produced by *A. flavus* in mycelia and culture medium were determined by the multi-analyte “dilute and shoot” LC-MS/MS method of Malachova *et al*.^50^. For the analysis of mycelia, 125 mg of the lyophilised mycelia were mixed with 1 mL of extraction solvent (acetonitrile/water/acetic acid 79:20:1, v/v/v) and extracted for 90 min at ambient temperature using GLF 3017 rotary shaker (GLF, Germany). After extraction, 500 µL of the extracts were transferred into glass vials and diluted with 500 µL of dilution solvent (acetonitrile/water/acetic acid 20:79:1, v/v/v). Vial contents were vigorously mixed and 5 µL was injected directly into the LC-MS/MS system. For the determination of metabolites in YES medium, a ten-fold dilution of 100 µL of the medium with mixture of extraction solvent and dilution solvent (1:1, v/v) in glass vials without any pre-treatment was performed.

The screening and detection of metabolites was performed as described by Malachová *et al*.^50^, and in brief the QTrap 5500 MS/MS detector (Applied Biosystems, Foster City, CA) equipped with TurboV electrospray ionization (ESI) source, and Agilent 1290 binary UHPLC system (Agilent Technologies, Waldbronn, Germany) were used. For the separation of the metabolites, the C18 security guard pre-column (4 × 3 mm i.d.) (Phenomenex, Torrance, CA, US) with the Gemini® C18 column (150 × 4.6 mm i.d., 5 µm particle size) was used. Freshly prepared eluents and the gradient were exactly as described by Malachová *et al*.^50^. The Scheduled selected reaction monitoring (sSRM) mode was applied, and two runs per sample were used (each for one mode). The detection window was set to ± 27 s in positive and ± 42 s in negative mode due to high number of monitored metabolites. The ESI source parameters were exactly as described by Malachová *et al*.^50^. At least two sSRM transitions were monitored per metabolite (quantifier and qualifier), and according to the validation guidelines the ratio between two transitions were used as additional identity confirmation point.

### Statistical analysis

Data are expressed as the mean value ± SEM from three separate experiments. The pooled datasets were checked for normality distribution by Shapiro-Wilk test and compared by nonparametric statistics methods (Friedman ANOVA and Kendall coefficient of concordance; Kruskal-Wallis ANOVA). The programme package Statistica 13.1 (Dell Inc., Texas, USA) was used and differences were considered significant when the *p* value was < 0.05. For the drawing of the Sankey diagrams Flourish studio was used (Flourish Studio, Kiln Enterprises Ltd, London, UK).
